# Evaluation on the presence of *Anaplasma*, *Ehrlichia*, and *Babesia* spp. in goats (*Capra hircus*) in Cebu, the Philippines

**DOI:** 10.14202/vetworld.2019.774-777

**Published:** 2019-06-11

**Authors:** Adrian P. Ybañez, Orgil V. Arrabis, Dennis Justin M. Alvarez, Eloiza May S. Galon, Rhea Mae P. Jayag, Elmie S. Delan, Rochelle Haidee D. Ybañez, Xuenan Xuan

**Affiliations:** 1 College of Science and Regional Center for Molecular Diagnostics and Research, University of the Philippines Cebu, Gorordo Avenue, Lahug, Cebu City 6000, Philippines; 2 Institute of Molecular Parasitology and Vector-borne Diseases at Main Campus and College of Veterinary Medicine at Barili Campus, Cebu Technological University, Cor. M. J. Cuenco Ave. and R. Palma St., Cebu City 6000, Cebu, Philippines; 3 National Research Center for Protozoan Diseases, Obihiro University of Agriculture and Veterinary Medicine, Obihiro City, Hokkaido, Japan

**Keywords:** *Anaplasma* spp, *Babesia* spp, *Ehrlichia* spp, peripheral blood smear examination, polymerase chain reaction, tick-borne diseases

## Abstract

**Background:**

Tick-borne diseases are caused by a wide variety of viruses, pathogens, and diseases. *Anaplasma*, *Ehrlichia*, and *Babesia* spp. are among the most known tick-borne pathogens in Asia. In the Philippines, these pathogens were already reportedly present in dogs and large ruminants, but no study has been reported yet evaluating their presence in goats.

**Aim:**

The present study aimed to evaluate the presence of *Anaplasma*, *Ehrlichia*, and *Babesia* spp. in goats in Cebu, the Philippines.

**Materials and Methods:**

A total of 100 blood samples from goats were collected in Cebu, the Philippines. Profile of sampled goats including age, body score, and sex was obtained. Peripheral blood smear examination and DNA extraction were performed. Nested polymerase chain reaction assay was used to evaluate the presence of *Anaplasma*, *Ehrlichia*, and *Babesia* spp.

**Results:**

None of the samples were found positive with *Anaplasma*, *Ehrlichia*, and *Babesia* spp. infection.

**Conclusion:**

Tested goats were negative with *Anaplasma, Ehrlichia*, and *Babesia* spp. and calls for continuous surveillance of these pathogens due to the reported detection of these pathogens in other livestock animals in the area.

## Introduction

Tick-borne diseases (TBDs), which include anaplasmosis, ehrlichiosis, and babesiosis, continue to threaten the global livestock industry. TBDs have caused great economic costs in the ruminant industry of some countries [1,2]. In South Africa, goats, sheep, and cattle infected with different TBDs have shown low productivity [3]. In other countries, *Anaplasma* [4], *Ehrlichia* [5], and *Babesia* spp. [6] have already been detected in goats. Farmers raise goats for their short generation time, high fertility rate, and suitability even in harsh environmental conditions [7]. However, though goats are easily raised, they are prominently infected by tick-borne pathogens. As these animals can be housed or tethered in areas at proximity with other livestock animals, they serve as a potential reservoir of these pathogens [8].

*Anaplasma* spp. are obligate intracellular bacteria which target the red and white blood cells, causing animal anaplasmosis [9]. *Anaplasma* spp. infection causes tick-borne fever with symptoms such as anorexia, dullness, high fever, and decreased milk yield [10]. On the other hand, *Ehrlichia* spp. are a group of Gram-negative, obligate intracellular bacteria that target white blood cells, causing monocytic, and granulocytic ehrlichiosis. *Ehrlichia* spp. infection is characterized by fever, headache, myalgia, and shaking chills [11]. Another pathogen is the *Babesia* spp., which is an apicomplexan protozoan that infects the erythrocytes of the vertebrate hosts, causing babesiosis. Symptoms of babesiosis may occur individually or in unison including inappetence, fever, lethargy, jaundice, coughing, nasal discharge, pale mucous membranes, diarrhea, and hemoglobinuria [12].

In the Philippines, the selected tick-borne pathogens have already been detected in cattle, water buffaloes, and dogs [13-16]. However, no study has been published about the evaluation of these pathogens in goats despite the presence of *Rhipicephalus* (*Boophilus*) *microplus* ticks, which can also infect goats [17]. Ybanez *et al*. [13] also confirmed that *R*. (*B*.) *microplus* infests cattle, which is known to transmit the selected tick-borne pathogens [18-20]. This may imply that goats could also be a reservoir for tick-borne pathogens, which could be transmitted to other livestock animals. Therefore, confirmation on the presence of these pathogens in goats is desirable. This study aimed to determine the presence of *Anaplasma*, *Ehrlichia*, and *Babesia* spp. in goats in Cebu, the Philippines.

## Materials and Methods

### Ethical approval

The procedures performed in this study were guided by the principles of animal welfare, the Animal Welfare Act of the Philippines (RA 8485) and Administrative Order No. 40 of the Bureau of the Animal Industry of the Philippines.

### Blood sample collection

A total of 100 goats were sampled from a farm in Poblacion, Barili, Cebu, and the Philippines. This farm serves as a supplier for the auction market as it sources out goats from the different part of the region. Profile of sampled goats including age, body score, and sex was obtained. 3 mL of the blood sample was collected from the jugular vein using a BD K3 EDTA Vacutainer^®^ tube (Becton, Dickinson and Company, Franklin Lakes, NJ, USA) and was stored at −20°C until DNA extraction. Peripheral blood smear examination (PBSE) was performed to evaluate the presence of the selected tick-borne pathogens. Complete blood count (CBC) was also performed.

### DNA extraction

DNA was extracted from the blood samples using a QIAamp DNA Mini Kit (QIAGEN, Valencia, CA, USA) following the company recommendation. After measuring their concentrations, the DNA samples were stored at −20°C until use.

### Nested polymerase chain reaction (nPCR)

A nPCR assay was used to detect the selected tick-borne pathogens. Screening for *Anaplasma*, *Ehrlichia*, and *Babesia* spp. was done using two primer pairs [21,22], as shown in Table-1. The nPCR was carried out in a final total volume of 10 µl composed of 4.3 µl DDW, 2 µl 5× PCR buffer, 1.0 µl of MgCl_2_ (25 mM), 0.15 µl of dNTPs (10 mM each), 0.25 µl each of the forward and reverse primers (0.22 µM/reaction), 0.05 µl of Taq DNA Polymerase (5U/µl) (Promega Corporation, USA), and 2 µl of DNA template. Double distilled water was used as DNA template for negative controls. Amplification cycles included initial denaturation at 95°C for 3 min, 35 cycles of denaturation at 95°C for 30 s, annealing at 56°C for 30 s, and extension at 72°C for 1 min followed by a final extension at 72°C for 10 min. The PCR amplicons were visualized using a 1.5% agarose gel stained with GelRed under the Ultraviolet transilluminator (UPV, CA, USA) [23].

**Table-1:** Nucleotide primers for *Anaplasma* spp., *Ehrlichia* spp., and *Babesia* spp. detection.

Primers	Nucleotide sequence	Amplicon size (bp)
FD1	CCGAATTCGTCGACAACAGAGTTTGATCCTGGCTCAG	1300
RP2	TTCAGCATTGTTCCATCGGCATTCGAACCTAQGQCCC	
EHR16SD	GGTACC (C/T) ACAGAAGAAGTCC	345
EHR16SR	TAGCACTCATCGTTTACAGC	
BABF	GCGATGGCCCATTCAAGTTT	146
BABR	CGCCTGCTGCCTTCCTTAGA	

### Data processing and analysis

The data from the survey instrument, PBSE, and PCR results were encoded by Microsoft Excel. Simple percentage and mean and standard deviation were used to analyze the data.

### Results and Discussion

Most of the sampled goats were 2 years old and below, female, and with body scores having thin to normal conformation (Table-2). All goats had no apparent clinical signs. CBC findings revealed normal hematological values (table not shown). PBSE revealed that all blood smears were negative for *Anaplasma*, *Ehrlichia*, and *Babesia* spp. On the other hand, PCR also revealed negative results for all the samples tested for the three pathogens.

**Table-2:** Profile of selected goats in Cebu, the Philippines (n=100).

Parameter	Frequency (%)
Age	
<1 years old	29
>1, <2	26
2 years old	24
3 years old	11
>4 years old	10
Mean=2.26	
SD=0.99	
Sex	
Male	6
Female	94
Body score	
Thin (2)	52
Ideal (3)	48
Mean=2.48	
SD=0.50	

SD=Standard deviation

The negative results may indicate the presumed absence of the three pathogens in the sampled goats. However, it is also possible that the goats might have had a low parasitemia level and were at a chronic stage of the disease [15] since no clinical signs were observed. Negative PBSE needs to be confirmed by PCR, as some studies suggested that samples negative for inclusion bodies in the blood smear can be positive using PCR [24]. Low-grade parasitemia is best detected using PCR assays [25]. However, all DNA samples were negative for the aforementioned pathogens regardless of the methods used.

The negative results of the detection of the selected pathogens can be affected by several factors. This can include the presence of a competent vector. An attempt was made to collect ticks during the sampling. However, tick infestation was not observed during sample collection. The population density of ticks could have been affected by the external environment, where climatic conditions greatly affect their distribution and abundance [26]. The sample collection period was conducted in the months of November to February on a farm located in a relatively mountainous area. These months are considerably cooler than the other months of the year in the Philippines. In warmer months, higher tick prevalence rates are usually reported in other countries [27,28]. The lack of tick vector in this study could have lowered the chances of the goats to acquire the pathogens.

The goats used in this study came from the different parts of the Central Visayas Region of the Philippines. Exposure to ticks, history of infestation and vaccines, domestication management from the previous farm, previous contact with other animals, and profiles of the parents were unknown. Therefore, even with the absence of ticks during collection, it cannot be assumed that there were no previous exposures. Moreover, the known presence of the competent vector *R*. (*B*.) *microplus* in the Philippines is a continuous threat to the goat industry since these ticks have been already reported to infest goats.

Another reason would be the domestication management of the goats. The tested goats were kept in enclosures elevated from the ground. The only contact the goats have with the outside environment would be their caretakers and the feeds they eat. Moreover, the location could have been a factor, as the area of the sampled goats was mountainous, which is known to have lesser tick infestation reports than those goats found in plateaus [29]. Finally, acquired immunity of the tested goats may have influenced the negative result, as the natural immune reaction of the body may have prevented the infection [30].

## Conclusion

*Anaplasma*, *Ehrlichia*, and *Babesia* spp. were not detected in the sampled goats in Cebu, the Philippines.

## Recommendations

Continuous monitoring and surveillance with larger area coverage is recommended to further evaluate the presence of these pathogens.

## Authors’ Contributions

APY and RHDY conceptualized the study and helped in finalizing the manuscript. OVA and DJMA collected the samples and helped in writing the manuscript. RMPJ and ESD helped in writing the manuscript. EMSG and XX helped in the molecular testing of the samples and provided valuable insights and support in the conduct of the study. All authors finally read and approved the final manuscript.
